# Cardiovascular and metabolic changes following 12 weeks of tobacco and nicotine pouch cessation: a Swedish cohort study

**DOI:** 10.1186/s12954-025-01195-y

**Published:** 2025-04-16

**Authors:** Peder af Geijerstam, Annelie Joelsson, Karin Rådholm, Fredrik H. Nyström

**Affiliations:** 1 Department of Health, Medicine and Caring Sciences, Faculty of Medicine and Health Sciences, Linköping University, 581 83 Linköping, Sweden; 2 Primary Care Center Cityhälsan Centrum, Norrköping, Östergötland County Sweden; 3 The George Institute for Global Health, University of New South Wales, Sydney, Australia

**Keywords:** Blood pressure, Body weight, Cholesterol, Nicotine pouches, Snus

## Abstract

**Objectives:**

Use of snus, including tobacco and nicotine pouches, is increasing in many countries. Nicotine increases blood pressure (BP) acutely, but the long-term effects of quitting the regular use of snus pouches are unknown. The aim was to evaluate the effects of snus cessation on home BP and markers of the metabolic syndrome.

**Methods:**

Volunteers aged 18–70 years using snus daily were invited to abruptly end their snus intake and followed using home BP and metabolic measurements before and for 12 weeks after cessation.

**Results:**

Fifty volunteers were recruited. Of these, 46 (92%) attempted snus cessation and 37 (74%) did not use snus for at least 3 weeks and were included in the study. Of those included, 33 maintained snus cessation for all 12 weeks. The mean age was 38 (± 10) years and 24 (65%) were men. At week 12, the mean changes in systolic home BP was 3.7 (95% CI 1.5–5.9) mmHg, in body weight was 1.8 (95% CI 1.1–2.4) kg, and in HbA1c was 0.7 (95% CI 0.0–1.6) mmol/mol.

**Conclusions:**

Cessation of tobacco and nicotine pouches in individuals who regularly used snus negatively impacted systolic home BP, body weight and HbA1c after 12 weeks. Whether these effects would be reversed by snus re-initiation cannot be determined by this study, but our novel findings suggest that successful cessation of regular snus usage does not immediate improve these cardiovascular risk factors. We call for further research to confirm our findings and evaluate the effects over longer time frames.

**Clinical Trial Registry number:**

NCT06019910, https://clinicaltrials.gov/study/NCT06019910.

**Supplementary Information:**

The online version contains supplementary material available at 10.1186/s12954-025-01195-y.

## Introduction

Hypertension is the leading risk factor for loss of disability-adjusted life-years, followed by smoking [1]. Tobacco smoke contains over 4000 potential toxicants, including nicotine, which is an addictive alkaloid and a natural insecticide of tobacco plants, *Nicotiana* spp. [2, 3]. Through effects on the nervous system and catecholamine release, nicotine acutely increases blood pressure (BP) and heart rate, peaking 5–10 min after exposure [2–5]. Over time, tolerance to nicotine reduces its hemodynamic effects, and the BP of individuals who smoke cigarettes is often lower than in individuals who do not smoke cigarettes [3, 6, 7]. However, the association over time may also depend on the time between smoking and BP measurement [5].

The prevalence of overweight and obesity, which is associated with type 2 diabetes, is also increasing [8]. Smoking increases energy expenditure and individuals who smoke cigarettes are less likely to be obese than individuals who do not smoke cigarettes, but results on long-term metabolic effects are conflicting [9–12]. Compared with individuals who do not smoke, individuals who smoke have a higher proportion of visceral fat, which is associated with insulin resistance, but smoking cessation is associated with weight gain [8–10]. The underlying mechanisms may include effects on energy expenditure, appetite, reward thresholds, and behaviors [9, 10].

Smokeless tobacco products are used by 6% of the global population [2]. Snus is administered sublabially and the nicotine is absorbed by the oral mucosa [13, 14]. Snus pouches may either include dry or moist tobacco, “tobacco pouches”, or only nicotine, binders and flavors, “nicotine pouches”, Supplementary Fig. 1. The use of nicotine pouches is increasing in both the UK and the US [15, 16]. Snus use is associated with several cardiovascular risk factors, including lower education, male sex, alcohol intake, cigarette smoking, and physical inactivity [13]. In prospective cohort studies, snus use is associated with incident cardiovascular disease and cancer, but results are conflicting and confounding may be difficult to fully adjust for [17, 18].

Tobacco pouches, but not nicotine pouches, are banned by the European Union since 1992, exempt Sweden [19]. The introduction and marketing of nicotine pouches is rapidly expanding both in and outside of Europe, but research is limited and independent data is insufficient according to several reports and studies, including the UK Committee on Toxicology [2, 14, 19]. Despite suggestions from both the scientific community and government agencies that nicotine may cause cardiometabolic harm long-term [18, 20], we know of no previously published trials that prospectively reported on beneficial cardiometabolic effects when quitting regular snus use. It was recently shown that nicotine in snus acutely increases BP, and both smoking and snus has been linked with increased risk for diabetes [4, 21]. Hence, the primary aim of this study was to evaluate the effects of snus cessation, and anticipated subsequent snus relapse, on home BP for up to 3 months. The secondary aim was to evaluate the effects on traditional markers of the metabolic syndrome and cardiovascular disease.

## Methods

The study had a non-randomized, prospective, observational cohort design. Based on a power calculation using the Clincalc.com (ClinCalc LLC, Arlington Heights, IL, USA) sample size calculator with an assumed average BP of 120 mmHg, a BP variability of 5 mmHg, a clinically relevant difference of 6 mmHg, an enrollment ratio of 3, an alpha of 0.05, and a power of 80%, at least 28 participants, of which at least 7 in each of the cessation and relapse groups, were to be included.

Post-hoc, because of the low number of participants in the relapse group and thus analysis of results in only the cessation group, the power of the study was calculated as 91.4% using the R package pwr, the function pwr.t.test, an alpha of 0.05, based on the mean and standard deviations of the difference in systolic home BP delta value between the run-in and week 12.

### Participants

Volunteers were recruited in Östergötland County, Sweden, via advertisement through social media and in public spaces, including a primary care center. Inclusion criteria were age 18 to 70 years, daily snus use (defined as at least once daily for at least 1 month), and the ability to use online questionnaires written and answered in Swedish. Exclusion criteria were simultaneous use of any other nicotine or tobacco product (including cigarettes, e-cigarettes, and nicotine replacement therapy), drug use (including cannabis), alcohol dependence, eating disorder, and current or planned pregnancy. Participants were reimbursed 1000 SEK (approximately 95 USD).

### Baseline measurements

Participants were booked for a baseline visit with the study investigators (a licensed nurse, AJ, or a medical doctor, PaG) for oral and written study information, acquirement of consent, and snus cessation support (Appendix 1).

Brachial office BP was measured bilaterally in the seated position using the validated oscillometric Omron M7 Intelli IT (Omron, Kyoto, Kyoto prefecture, Japan) device. The right arm was designated as the reference arm for further measurements unless the BP was > 10 mmHg higher in the left arm. Height was measured using a wall-mounted telescopic measure and recorded in centimeters with one decimals precision.

At the laboratory, fasting serum creatinine, plasma sodium, plasma potassium, plasma glucose, blood glycated hemoglobin (HbA1c), serum insulin, plasma triglycerides, plasma lipid profile (total cholesterol, low-density lipoprotein [LDL], high-density lipoprotein [HDL], and non-high density lipoprotein [non-HDL]), and plasma hsCRP, were taken, and in light clothing without shoes, body weight in kg with one decimals precision was recorded.

A baseline questionnaire included past and current nicotine and tobacco use, cardiovascular disease amongst first-degree relatives, the Alcohol use disorders identification test (AUDIT), a translated version of the short-form food frequency questionnaire (SFFFQ) [22], the validated questionnaire on physical activity from the Swedish National Board of Health and Welfare [23], as well as the validated SED-GIH questionnaire of The Swedish School of Sport and Health Sciences on sedentary behavior [24].

For 3 days, participants also recorded their daily snus use as well as measured their home BP with the same BP device as above, 3 times in the morning and 3 times in the evening. Participants were instructed to perform each of the 3 consecutive measurements with at least 1 min between each.

Participants then selected a date within the next 7 days, but no earlier than the day after the third day of baseline BP measurements, on which to abruptly end their snus intake.

### Follow-up measurements

As long as participants did not use any nicotine, collection of body weight, blood samples (fasting plasma glucose, blood HbA1c, serum insulin, plasma lipid profile, and plasma hsCRP), AUDIT and the SFFFQ were repeated at the end of weeks 4 and 12. If participants resumed daily snus intake (defined as at least 1 pouch per day for at least 3 days), body weight, blood samples, AUDIT and the SFFFQ were repeated no later than the following weekday, as well as after 4 and 12 weeks of relapse. If participants started using any other nicotine product (as specified in the exclusion criteria), they were withdrawn from the study. During the entire study, regardless of snus cessation or snus relapse, participants continued to measure their home BP daily, 3 times in the morning and 3 times in the evening, or as often as possible.

All study data were collected through the digital platform REDCap versions 13.7.19 to 14.0.29 (Vanderbilt University, Nashville, TN, USA).

### Statistical analyses

Number of pack years was calculated as the number of consumption years multiplied by the number of cigarettes per day, divided by 20. Number of pouch years was calculated as the number of consumption years multiplied by the average number of pouches per day. Consumption durations between 0 and 1 year was recorded as 0.5 years. Mean daily nicotine consumption during the run-in period was calculated as the mean number of snus pouches per day multiplied by the pouch nicotine content.

The changes in systolic and diastolic BP and heart rate, between the run-in period and weeks 1 to 12 of snus cessation were tested using a paired 2-samples *t* test and presented as the means and 95% CI for each period, as well as the differences between them. The changes in body weight, blood sample values, the SFFFQ scores and the AUDIT score were tested similarly, and presented for the run-in period, as well as weeks 4 and 12.

In subgroup analyses of change in body weight and blood sample values, linear regression was used to calculate the change between the run-in period, week 4 and week 12 for participants using nicotine vs tobacco pouches during the run-in period. Values were presented crude and adjusted for age, sex, and pouch years.

Missing data was managed by listwise deletion. Data analyses were made using R version 4.4.0 (R Core Team, Vienna, Austria) and RStudio version 2024.04.1 + 748 (Posit Software, Boston, MA, USA). Statistical tests were 2-tailed and *P* values < 0.05 were considered statistically significant.

### Ethical

The study complied with the declaration of Helsinki and was approved by the Swedish Ethical Review Authority (Dnr 2023-03376-01, 2023-06640-02, and 2024-01128-02). All participants gave written, informed consent prior to participation. Prior to commencement, the study was registered at ClinicalTrials.gov (registration number NCT06019910).

## Results

Between October 9th, 2023, and March 4th, 2024, 50 volunteers were recruited, and data collection concluded on May 31st, 2024. Of participants, 4 (8%) were lost to follow-up before snus cessation and the remaining 46 (92%) attempted snus cessation. Of these, 9 (out of which 8 resumed snus intake) withdrew before the 3rd week, Supplementary Fig. 2.

The remaining 37 (74%) did not use snus for at least 3 weeks and were included in the study. Of these, 1 resumed snus intake and continued the study in the relapse group, 1 started smoking cigarettes and was excluded, 2 resumed snus intake, but withdrew, and 33 (72% of the 46 whom attempted snus cessation and 89% of the 37 included participants) did not use snus for all 12 weeks. Because of low power (n = 1), the effects of snus relapse were not analyzed.

Of participants, 24 (65%) were men and the mean age was 38 (± 10) years, Table 1. The mean systolic and diastolic home BP was 114.3 and 72.5 mmHg. Current use of tobacco and nicotine pouches was reported by 25 (68%) and 13 (35%) participants with a median of 233 and 24 pouch years, respectively. During the run-in, 24 (65%) used tobacco pouches, 12 (32%) used nicotine pouches, and 1 (3%) used both. During the run-in period, participants consumed a median of 144.3 mg of nicotine daily, and participant that used tobacco vs nicotine pouches had a higher intake, median (Q1–Q3) 155 (124–205) vs 64 (36–112) mg per day, *P* < 0.001, Supplementary Fig. 3A.

**Table 1:** Baseline characteristics

	All participants, N = 37	Men, n = 24	Women, n = 13
*Age (years), mean (SD)*	38 (10)	38 (11)	40 (10)
*Medications*			
Beta-blockers	1 (3)	1 (4)	0
Lipid- or glucose-lowering medications	0	0	0
*Morbidities of first-degree relatives*			
Hypertension	12 (32)	6 (25)	6 (46)
Diabetes	5 (14)	2 (8)	3 (23)
Cardiovascular disease	8 (22)	6 (25)	2 (15)
*Weekly physical exercise with breathlessness*			
None	4 (11)	1 (4)	3 (23)
Less than 30 min	2 (5)	1 (4)	1 (8)
30–60 min	7 (19)	3 (13)	4 (31)
60–90 min	6 (16)	3 (13)	3 (23)
90–120 min	2 (5)	1 (4)	1 (8)
More than 120 min	16 (43)	15 (63)	1 (8)
*Weekly physical exercise without breathlessness*			
None	0	0	0
Less than 30 min	1 (3)	0	1 (8)
30–60 min	10 (27)	7 (29)	3 (23)
60–90 min	6 (16)	5 (21)	1 (8)
90–150 min	6 (16)	3 (13)	3 (23)
150–300 min	8 (22)	4 (17)	4 (31)
More than 300 min	6 (16)	5 (21)	1 (8)
*Daily time in the seated position*			
Almost the entire day	2 (5)	0	2 (15)
13–15 h	0	0	0
10–12 h	13 (35)	10 (42)	3 (23)
7–9 h	17 (46)	10 (42)	7 (54)
4–6 h	3 (8)	3 (13)	0
1–3 h	2 (5)	1 (4)	1 (8)
None	0	0	0
*AUDIT (score)*	2 (1–3)	2 (1–3)	1 (0–2)
*Past cigarette use*			
Combustion cigarettes	15 (41)	6 (25)	9 (69)
Combustion cigarettes (pack years)^a^	6.0 (1.3–11.3)	5.0 (0.9–13.1)	6.0 (2.0–11.3)
Electronic cigarettes	1 (3)	0	1 (8)
Electronic cigarettes (pack years)^a^	2.0 (2.0–2.0)	0	2.0 (2.0–2.0)
*Tobacco pouches use*			
Never	5 (14)	2 (8)	3 (23)
Previous	7 (19)	4 (17)	3 (23)
Current	25 (68)	18 (75)	7 (54)
Pouch years^a^	233 (160–400)	257 (180–400)	200 (100–319)
*Nicotine pouches use*			
Never	22 (60)	17 (71)	5 (39)
Previous	2 (5)	1 (4)	1 (8)
Current	13 (35)	6 (25)	7 (54)
Pouch years^a^	24 (11–35)	15 (8–22)	35 (24–69)
*Snus during run-in, product type*			
Nicotine pouches	12 (32)	6 (25)	6 (46)
Tobacco pouches dry	15 (41)	10 (42)	5 (39)
Tobacco pouches moist	7 (19)	7 (29)	0
Both moist and dry tobacco pouches	2 (5)	1 (4)	1 (8)
Both nicotine and tobacco pouches	1 (3)	0	1 (8)
*Snus during run-in, product content*			
Nicotine per pouch (mg)	8.0 (6.8–11.6)	9.0 (7.5–12.7)	8.0 (5.6–8.4)
Any product with licorice flavor	10 (27)	4 (17)	6 (46)
*Snus during run-in, consumption*			
Pouches per day	16 (11–22)	18 (10–23)	16 (14–20)
Nicotine per day (mg)	144.3 (98.3–190.0)	152.0 (104.3–204.7)	117.3 (57.6–153.5)
*Lifetime snus nicotine intake (g)*^b^	723.5 (210.7–1296.4)	949.1 (582.9–1607.0)	436.7 (150.6–976.5)
*Body mass index (kg/m*^*2*^*)*	26.7 (23.3–28.6)	26.5 (23.6–28.3)	26.7 (22.9–35.5)
*Laboratory results*			
Plasma creatinine (µmol/L)	81 (73–89)	87 (81–91)	72 (65–78)
Serum sodium (mmol/L)	140 (140–141)	140 (140–141)	141 (140–141)
Serum potassium (mmol/L)	4.2 (4.0–4.3)	4.2 (4.1–4.4)	3.9 (3.9–4.2)
Plasma total cholesterol (mmol/L)	4.9 (4.2–5.5)	5.0 (4.2–5.6)	4.8 (4.3–5.3)
Plasma triglycerides (mmol/L)	1.1 (0.8–1.4)	1.1 (0.7–1.4)	1.0 (0.9–1.1)
Plasma LDL (mmol/L)	2.9 (2.3–3.5)	2.9 (2.3–3.4)	2.9 (2.2–3.5)
Plasma HDL (mmol/L)	1.3 (1.2–1.6)	1.2 (1.1–1.7)	1.3 (1.2–1.5)
Plasma non-HDL (mmol/L)	3.6 (2.7–4.0)	3.6 (2.9–3.9)	3.6 (2.6–4.0)
Plasma hsCRP (mg/L)	0.7 (0.3–1.4)	0.4 (0.3–0.8)	1.4 (0.5–2.4)
Plasma glucose (mmol/L)	5.1 (5.0–5.6)	5.2 (5.0–5.6)	5.0 (4.9–5.5)
Blood glycated hemoglobin (mmol/mol)	36 (33–37)	35 (33–37)	36 (34–37)
Plasma insulin (mIE/L)	6.6 (4.5–12.0)	6.4 (4.4–10.3)	11.0 (5.8–12.0)
*BP measurements, mean (SD)*			
Office BP, systolic (mmHg)	117.3 (11.7)	121.1 (11.2)	110.2 (9.2)
Office BP, diastolic (mmHg)	74.1 (9.8)	73.0 (10.9)	76.0 (7.5)
Office heart rate (beats per minute)	69.5 (10.5)	67.6 (11.3)	73.1 (8.0)
Home BP, systolic (mmHg)	114.3 (12.3)	118.7 (10.9)	106.2 (10.6)
Home BP, diastolic (mmHg)	72.5 (7.4)	71.9 (7.9)	73.4 (6.8)
Home heart rate (beats per minute)	67.5 (9.0)	64.8 (9.0)	72.3 (6.9)
*Relapsed during study*	4 (11)	3 (13)	1 (8)

Categorical variables are in numbers and percentages and continuous variables in median and interquartile range, unless otherwise stated

*AUDIT* The Alcohol Use Disorders Identification Test, *BP* blood pressure, *HDL* high-density lipoprotein, *hsCRP* high-sensitivity C-reactive protein, *LDL* low-density lipoprotein

^a^Cigarette pack years were calculated as the years of consumption multiplied by the number of cigarettes per day, divide by 20. Snus pouch years were calculated as the years of consumption multiplied by the average number of pouches per day. Years of consumption less than 1 was counted as 0.5

^b^Lifetime snus nicotine consumption in grams was calculated as the sum of the number of consumption years of nicotine and tobacco snus multiplied by 365.4 days multiplied by the mean nicotine consumption during the 3-day run-in

**Fig. 1:**
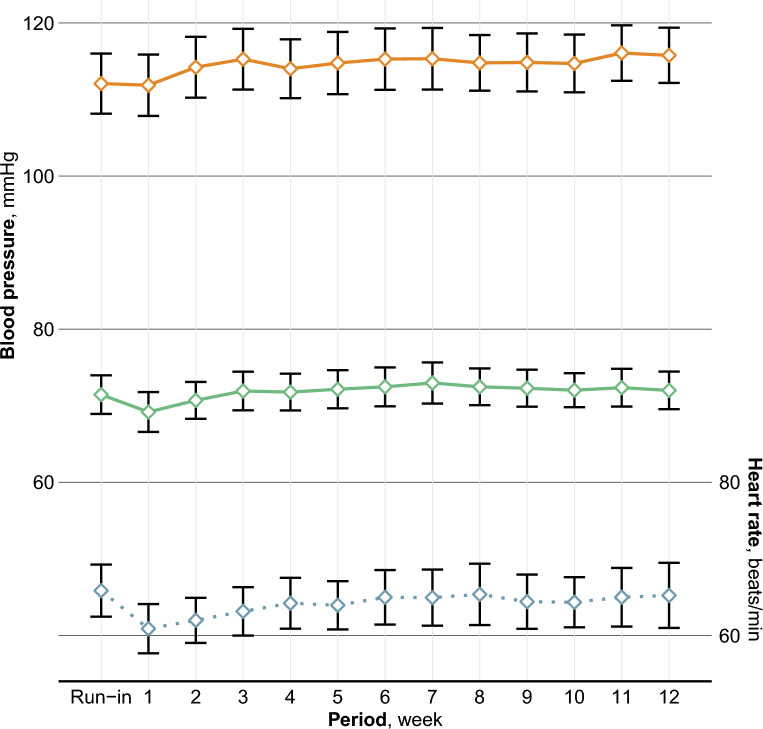
Systolic (top) and diastolic (middle) blood pressure and heart rate (bottom) presented as the mean and 95% confidence intervals during the run-in and each week of snus cessation

The systolic home BP increased with a mean 3.7 (95% CI 1.5–5.9) mmHg until week 12, beginning from the 5th week, Table 2 and Fig. 1. The diastolic home BP decreased by a mean 2.5 (95% CI 1.3–3.8) mmHg during the first week, but this change was attenuated during week 2 and no longer significant from week 3. The heart rate decreased by a mean 5.7 (95% CI 3.9–7.6) beats/minute during the first week, and to a lesser degree during weeks 2 to 7, after which it was no longer different from that of the run-in period.

**Table 2:** Systolic and diastolic blood pressure and heart rate during the run-in and each week of snus cessation presented as the mean and 95% confidence interval

	N	Systolic BP (mmHg)	Change vs run-in	Diastolic BP (mmHg)	Change vs run-in	Heart rate (beats/minute)	Change vs run-in
		Mean (95% CI)	Mean (95% CI)	Mean (95% CI)	Mean (95% CI)	Mean (95% CI)	Mean (95% CI)
Run-in	37	114.3 (110.2–118.4)	0 ref	72.5 (70.0–74.9)	0 ref	67.5 (64.5–70.5)	0 ref
Week 1	37	113.8 (109.7–117.9)	− 0.5 (− 2.0 to 1.1)	69.9 (67.3–72.5)	− 2.5 (− 3.8 to − 1.3)	61.7 (58.9–64.6)	− 5.7 (− 7.6 to − 3.9)
Week 2	36	115.8 (111.7–119.8)	1.1 (− 0.6 to 2.8)	71.3 (68.9–73.8)	− 1.6 (− 3.0 to − 0.1)	62.9 (60.1–65.7)	− 5.1 (− 6.9 to − 3.2)
Week 3	36	116.6 (112.5–120.8)	2.0 (− 0.1 to 4.1)	72.2 (69.6–74.8)	− 0.7 (− 2.3 to 1.0)	64.0 (61.2–66.8)	− 4.0 (− 5.8 to − 2.1)
Week 4	36	116.1 (111.7–120.5)	1.5 (− 0.6 to 3.5)	72.3 (69.7–74.8)	− 0.6 (− 2.3 to 1.1)	64.6 (61.8–67.4)	− 3.4 (− 5.4 to − 1.3)
Week 5	34	117.1 (112.5–121.6)	2.5 (0.3 to 4.7)	73.0 (70.3–75.7)	0.3 (− 1.2 to 1.8)	64.5 (61.7–67.4)	− 2.8 (− 4.3 to − 1.3)
Week 6	32	117.6 (113.1–122.0)	2.4 (0.3 to 4.6)	73.2 (70.4–76.0)	0.4 (− 1.4 to 2.2)	65.1 (61.9–68.4)	− 1.9 (− 3.4 to − 0.3)
Week 7	32	117.7 (113.2–122.1)	2.5 (0.3 to 4.8)	73.4 (70.6–76.2)	0.7 (− 1.1 to 2.5)	65.2 (61.9–68.6)	− 1.8 (− 3.2 to − 0.4)
Week 8	31	116.1 (112.5–119.6)	2.0 (− 0.1 to 4.0)	72.7 (70.2–75.3)	0.6 (− 1.4 to 2.5)	65.4 (61.7–69.1)	− 1.4 (− 3.3 to 0.5)
Week 9	31	115.5 (111.9–119.2)	2.3 (0.2 to 4.4)	71.9 (69.5–74.3)	0.4 (− 1.5 to 2.3)	64.3 (60.9–67.7)	− 1.6 (− 3.4 to 0.1)
Week 10	30	115.0 (111.3–118.7)	2.5 (0.4 to 4.5)	71.6 (69.4–73.9)	0.5 (− 1.3 to 2.2)	64.0 (60.8–67.2)	− 1.7 (− 3.4 to − 0.0)
Week 11	28	116.1 (112.5–119.7)	3.4 (1.2 to 5.7)	72.4 (69.9–74.8)	0.7 (− 1.3 to 2.7)	65.0 (61.2–68.8)	− 0.4 (− 2.1 to 1.3)
Week 12	29	115.8 (112.2–119.4)	3.7 (1.5 to 5.9)	72.0 (69.5–74.5)	0.6 (− 1.6 to 2.7)	65.2 (61.0–69.5)	− 0.6 (− 2.8 to 1.5)

Differences between each week of snus cessation and the run-in were made using a 2-sided paired samples *t* test and presented as the mean (95% CI) difference

*N* number of participants with valid measurements

**Fig. 2:**
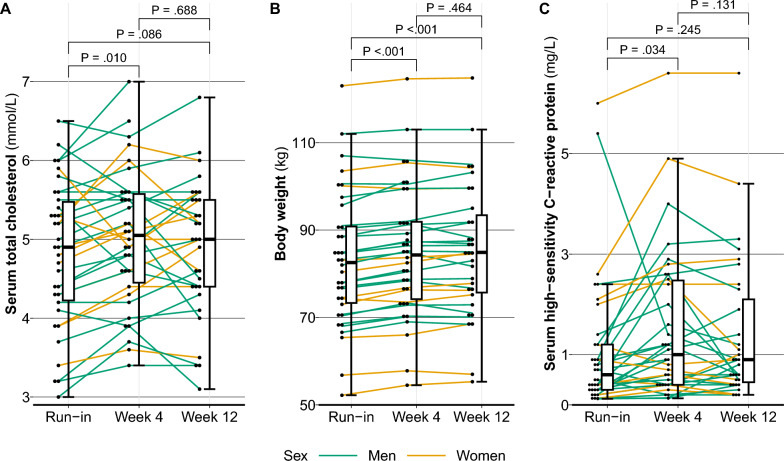
Serum total cholesterol (**A**), body weight (**B**), and high-sensitivity C-reactive protein (**C**) during run-in, at week 4, and at week 12. Missing values for week 4 (n = 1 for serum total cholesterol, n = 2 for body weight, and n = 2 for serum high-sensitivity C-reactive protein) were interpolated as the sum of the value of the run-in and week 12 divided by 2. Comparisons were made using a paired samples 2-way t test. For plot **C**, one outlier during week 4 of 23.0 mg/L was removed because of suspected acute transitory illness

From the run-in period to week 4, participants’ body weight increased with a mean 1.8 (95% CI 1.4–2.3) kg, and this increase remained until week 12, and plasma cholesterol levels increased with 0.22 (95% CI 0.05–0.38) mmol/L, but this was attenuated at week 12, Table 3 and Fig. 2. Both plasma HDL and non-HDL contributed to the increase in total cholesterol. Blood HbA1c levels had increased at week 12 with 0.7 (95% CI 0.0–1.6) mmol/mol, and plasma hsCRP levels had increased at week 4 with 0.46 (95% CI 0.03–0.88) mg/L, but this difference was no longer significant at week 12, 0.25 (95% CI − 0.18 to 0.68) mg/L. No changes were observed in the food and alcohol consumption questionnaires, Supplementary Table 1.

**Table 3:** Mean and 95% confidence intervals of cardiometabolic measurements for participants during snus cessation

	N	Run-in	Week 4	Week 12	Change	
		Mean (95% CI)	Mean (95% CI)	Mean (95% CI)	Mean (95% CI)	*P*
*Body weight (kg)*						
Run-in to Week 4	32	82.7 (77.1–88.2)	84.5 (79.0–90.0)		1.8 (1.4 to 2.3)	< .001
Week 4 to Week 12	29		84.5 (78.6–90.4)	84.7 (78.8–90.6)	0.2 (− 0.4 to 0.8)	.555
Run-in to Week 12	31	83.5 (77.7–89.3)		85.3 (79.6–91.0)	1.8 (1.1 to 2.4)	< .001
*Plasma total cholesterol (mmol/L)*						
Run-in to Week 4	33	4.79 (4.46–5.12)	5.01 (4.70–5.32)		0.22 (0.05 to 0.38)	.010
Week 4 to Week 12	30		4.90 (4.61–5.20)	4.87 (4.54–5.19)	− 0.04 (− 0.22 to 0.15)	.688
Run-in to Week 12	31	4.72 (4.39–5.05)		4.89 (4.57–5.21)	0.17 (− 0.03 to 0.37)	.086
*Plasma triglycerides (mmol/L)*						
Run-in to Week 4	33	1.03 (0.91–1.15)	1.09 (0.93–1.25)		0.06 (− 0.06 to 0.18)	.288
Week 4 to Week 12	30		1.06 (0.91–1.22)	1.03 (0.81–1.24)	− 0.04 (− 0.17 to 0.10)	.587
Run-in to Week 12	31	1.12 (0.90–1.33)		1.09 (0.85–1.34)	− 0.03 (− 0.18 to 0.13)	.732
*Plasma LDL (mmol/L)*						
Run-in to Week 4	33	2.90 (2.63–3.17)	3.04 (2.79–3.28)		0.13 (− 0.01 to 0.27)	.064
Week 4 to Week 12	30		3.01 (2.74–3.28)	2.99 (2.70–3.28)	− 0.02 (− 0.20 to 0.16)	.819
Run-in to Week 12	31	2.85 (2.58–3.13)		3.00 (2.72–3.28)	0.15 (− 0.04 to 0.33)	.124
*Plasma HDL (mmol/L)*						
Run-in to Week 4	33	1.41 (1.26–1.56)	1.47 (1.31–1.63)		0.06 (0.02 to 0.09)	.002
Week 4 to Week 12	30		1.40 (1.26–1.55)	1.39 (1.23–1.54)	− 0.02 (− 0.07 to 0.03)	.452
Run-in to Week 12	31	1.34 (1.21–1.48)		1.37 (1.22–1.52)	0.03 (− 0.02 to 0.09)	.236
*Plasma non-HDL (mmol/L)*						
Run-in to Week 4	33	3.37 (3.07–3.67)	3.54 (3.27–3.81)		0.17 (0.02 to 0.32)	.025
Week 4 to Week 12	30		3.50 (3.21–3.79)	3.47 (3.16–3.77)	− 0.03 (− 0.21 to 0.14)	.702
Run-in to Week 12	31	3.36 (3.04–3.69)		3.50 (3.20–3.81)	0.14 (− 0.04 to 0.32)	.124
*Plasma glucose (mmol/L)*						
Run-in to Week 4	33	5.23 (5.07–5.39)	5.24 (5.09–5.39)		0.01 (− 0.11 to 0.13)	.840
Week 4 to Week 12	30		5.23 (5.07–5.39)	5.31 (5.11–5.51)	0.08 (− 0.02 to 0.18)	.104
Run-in to Week 12	31	5.24 (5.06–5.41)		5.31 (5.12–5.50)	0.08 (− 0.06 to 0.21)	.255
*Blood HbA1c (mmol/mol)*						
Run-in to Week 4	33	34.9 (33.9–36.0)	34.9 (33.8–36.0)		0.0 (− 0.6 to 0.6)	.919
Week 4 to Week 12	30		35.0 (33.8–36.2)	35.6 (34.4–36.9)	0.6 (− 0.1 to 1.5)	.097
Run-in to Week 12	31	35.0 (33.9–36.1)		35.7 (34.5–37.0)	0.7 (0.0 to 1.6)	.048
*Plasma insulin (mIE/L)*						
Run-in to Week 4	33	8.06 (6.56–9.55)	8.58 (6.84–10.32)		0.52 (− 0.59 to 1.63)	.343
Week 4 to Week 12	30		8.84 (6.95–10.72)	8.60 (6.58–10.63)	− 0.23 (− 1.60 to 1.14)	.730
Run-in to Week 12	31	8.69 (6.95–10.44)		8.81 (6.81–10.81)	0.12 (− 1.28 to 1.51)	.866
*Plasma hsCRP (mg/L)*						
Run-in to Week 4	32	1.02 (0.52–1.51)	1.47 (0.92–2.02)		0.46 (0.03 to 0.88)	.036
Week 4 to Week 12	29		1.52 (0.92–2.13)	1.33 (0.77–1.89)	− 0.19 (− 0.43 to 0.05)	.116
Run-in to Week 12	31	1.16 (0.64–1.69)		1.41 (0.88–1.95)	0.25 (− 0.18 to 0.68)	.245

Differences between each week of snus cessation and the run-in were made using a 2-sided paired samples *t* test and presented as the mean (95% CI) difference

*N* number of participants with valid measurements

## Discussion

To the best of our knowledge, this is the first prospective study on the cardiometabolic effects of snus cessation. We found a relatively high snus cessation rate, and that cessation was associated with increased systolic BP and body weight, as well as transient increases in both plasma hsCRP and cholesterol levels.

### Cessation rate

The 74% 12-week snus cessation rate was comparatively high. For cigarette smoking, a 2017 Cochrane review showed a 3–6 month cessation rate of 11% after individual counselling [25]. For snus, a 2015 Swedish study showed a 3-month cessation rate of 4%, and advice received at the dentistry clinic by 50% of the participants did not affect the outcome [26]. The markedly higher cessation rate in our study could be explained by a participation bias of individuals that were highly motivated and/or the rigorous follow-up using home BP measurements and blood samples. Our study had the explicit aim to assess the effects of snus cessation, which is different from the 2015 Swedish study on snus cessation. The different cessation rate to studies of smoking cessation could also relate to the slower release of snus nicotine [19]. A 2023 cross-over study found that maximum plasma nicotine concentrations were reached earlier, and was higher, after cigarette smoking vs nicotine pouch use, and that cigarette smoking better relieved craving [27]. Results on peak plasma nicotine concentration were similar in a 2024 cross-over study when analyzing nicotine pouches with 6 or 20 mg of nicotine, except that the use of nicotine pouches with 30 mg of nicotine resulted in a higher peak than cigarette smoking [28]. This could indicate that cigarette smoking is more addictive and thus difficult to stop using. Studies randomizing participants to a follow-up protocol akin to ours vs no such support could clarify the effects of the rigorous follow-up.

### Blood pressure

Systolic BP increased markedly after snus cessation and did not decrease with time. In a 2024 cross-over study, the use of nicotine pouches containing 6 mg of nicotine increased systolic and diastolic BP within 6–20 min of exposure, but it was no longer different from baseline after 40 min even after use of the most potent pouches containing 30 mg of nicotine [28]. In the same study, the results were comparable to those of cigarette smoking [28]. Both combustion and electronic cigarettes are also known to increase systolic and diastolic BP within minutes to hours of exposure, but individuals who smoke cigarettes have lower BP compared to those who do not smoke [6, 29]. In an observational study with a follow-up of 4 years, cigarette cessation was associated with increased systolic and diastolic BP and incident hypertension > 1 year after cessation, but not prior [30].

A period effect such as procedure fatigue, with an increasingly relaxed stance towards the rigid measurement protocol, could have affected our results. However, in one study of home BP, the magnitude of the period effect was only around 1 mmHg after 3 weeks, and affected both the systolic and diastolic BP [31]. Because of the non-randomized, non-blinded study design, direct causality between nicotine cessation and BP increase cannot be established. Changes may also be mediated by e.g., weight gain or lifestyle changes.

Finally, we observed a transient reduction in heart rate after snus cessation, which is in line with nicotine cessation, given that nicotine use is known to increase the heart rate [2–5].

### Body weight

Body weight increased with 1.8 kg within the first 4 weeks of snus cessation which remained until 12 weeks, and the increase was numerically more pronounced for women. Smoking cessation is also associated with weight gain, usually around 3–5 kg, and mostly during the first months [9, 10]. In a study of 72 participants, 17 women who quit cigarette smoking did not gain weight during the first 35 days when they used nicotine patches, but gained 2.1 kg after another 42 days using neither cigarettes nor nicotine patches [32]. This indicates that nicotine may be responsible for this weight gain, possibly by reversed nicotine-related increases in energy expenditure, appetite-suppressant effects, or alterations of reward thresholds and behaviors [9–11]. Future studies may benefit from including additional biomarkers such as glucagon-like peptide 1, ghrelin and leptin, or indirect calorimetry measurements.

### Blood lipid levels

Plasma cholesterol levels increased after 4 weeks, remained numerically higher after 12 weeks, and was only seen in individuals using nicotine vs tobacco pouches in subgroup analyses. Total cholesterol and LDL levels are higher, and HDL levels are lower, in individuals who smoke cigarettes and chew tobacco compared with those who do not [33]. Cigarette smoking is associated with higher triglycerides and lower HDL levels, but nicotine replacement therapy does not affect triglycerides levels and results on HDL levels are conflicting [34]. HDL levels normalize with smoking cessation, but not when nicotine replacement therapy is used [32, 35]. Together with our results, this indicates a direct or indirect influence of nicotine on serum lipid levels. However, randomized controlled trials are needed to draw conclusions on causality.

### Markers of dysglycemia

Snus cessation was associated with increased HbA1c, but only in women participants in subgroup analyses. A study of 14 healthy volunteers found that serum cortisol was elevated after intake of snus with vs without nicotine, but no postprandial effects were seen on glucose [4]. Our results could be mediated by the increase in body weight, which was evident already at 4 weeks, and thus the remaining study duration was enough to affect HbA1c values which are related to the erythrocyte turnover of around 120 days. If so, HbA1c levels could be even higher with a longer follow-up.

### Inflammation

An increase in hsCRP of 0.5 mg/L was seen after 4 weeks, but this was largely normalized after 12 weeks, and in subgroup analyses only seen in participants using tobacco pouches during the run-in period. In individuals who smoke cigarettes, inflammatory markers, including CRP, are higher, and could be a link to cardiovascular disease [35–37]. Smoking is related to CRP levels in a dose–response manner, and smoking cessation reduces CRP levels, but only after at least 1 year [38, 39]. Individuals who use electronic cigarettes, however do not have higher CRP levels than those who do not smoke [37]. Nicotine may increase levels of inflammatory markers, including hsCRP, but may also have anti-inflammatory effects [37, 40]. Our results indicate that nicotine cessation may increase CRP levels transiently. Future studies may evaluate these changes on a longer time frame and include questionnaires on mood changes to understand whether these may be associated with CRP levels.

### Further research

Interpreting data on snus requires considerations of the differences between tobacco and nicotine pouches, moist and dry tobacco pouches, synthetic vs tobacco-derived nicotine, and variations in nicotine content between products. Salt and licorice content in snus products could also negatively affect BP and cardiovascular health [5]. Further research is also needed to better understand the potential effects of simultaneous consumption of foods and drinks [19]. It would also be valuable to determine the effects of snus in snus naïve individuals, especially long-term, but ethical challenges remain because of its addictive nature. Although our study shows that in motivated individuals receiving medium-term follow-up, snus cessation may be less challenging than expected, our data does not inform us on the long-term cessation rate.

### Limitations

The study has some important limitations. The trial was not randomized and controlled, and thus causality cannot be determined, partly because of an unexpectedly high cessation rate which resulted in a control group without sufficient power. Social desirability bias, such as a tendency for participants who relapse to leave the study, may have affected results. However, we informed participants that results were equally valuable regardless of snus cessation or relapse. Research participation effect may limit the external validity of the results to individuals who are interest to participate in a study with the explicit goal of snus cessation. We did not adjust for multiple comparisons, as suggested by Rothman [41], and thus the risk of type-I-errors needs to be considered in the interpretation of the results. Because the sample size calculation was based on home BP, results of other measurements may be underpowered and not used to rule out an effect. Finally, our study did not include measurements of cotinine, a nicotine metabolite, to confirm intake and cessation, respectively. However, any effect during the study from participants resuming snus use would likely have attenuated the results and affected the trend in, e.g., home BP. The participants were aware that they participated in a trial that would be published, and this might have increased cessation rates compared to a real-life setting. However, that is not different from many other trials on smoking cessation in which relapse has been far more common compared to our trial. The study was limited to individuals living in Sweden with an interest to stop using snus, and the external validity beyond this cohort is uncertain. Finally, whether snus relapse is harmful or not cannot be determined by our study because of the high cessation rate. Considering the rapidly growing market, widespread regulatory debates, and the importance to reduce the harm of cigarette smoking without introducing new harm, it is imperative that additional research answers questions including the mechanisms behind our findings, as well as the more long-term effects of both snus use and snus cessation.

## Conclusions and recommendations

Tobacco and nicotine pouch cessation was associated with increased BP and body weight for up to three months. Individuals at risk of hypertension or dysglycemia may need to be monitored during cessation, and further research is needed to evaluate the effects on a longer time frame. Importantly, whether these effects would be reversed by reinitiation of snus could not be determined by this study. Finally, the current findings need to be replicated in future studies to confirm their validity and generalizability.

## Supplementary Information

Additional file 1.

## Data Availability

The data that support the findings of this study are available on request from the corresponding author. The data are not publicly available due to privacy or ethical restrictions.

## Abbreviations

AUDIT: Alcohol use disorders identification test
BMI: Body mass index
BP: Blood pressure
eGFR: Estimated glomerular filtration rate
FFQ: Food frequency questionnaire
HbA1c: Glycated hemoglobin
HDL: High-density lipoprotein
hsCRP: High-sensitivity C-reactive protein

## References

[1] GBD. Risk Factor Collaborators: global, regional, and national comparative risk assessment of 84 behavioural, environmental and occupational, and metabolic risks or clusters of risks for 195 countries and territories, 1990–2017: a systematic analysis for the Global Burden of Disease Study 2017. Lancet. 2017;2018(392):1923–94. 10.1016/S0140-6736(18)32225-6 PMC6227755 30496105

[2] Dorotheo EU, Arora M, Banerjee A, Bianco E, Cheah NP, Dalmau R, Eissenberg T, Hasegawa K, Naidoo P, Nazir NT, et al. Nicotine and cardiovascular health: when poison is addictive—a WHF policy brief. Glob Heart. 2024;19:14. 38312998 10.5334/gh.1292 PMC10836189

[3] Middlekauff HR, Park J, Moheimani RS. Adverse effects of cigarette and noncigarette smoke exposure on the autonomic nervous system: mechanisms and implications for cardiovascular risk. J Am Coll Cardiol. 2014;64:1740–50. 25323263 10.1016/j.jacc.2014.06.1201

[4] Ismail M, Stagling S, Lundberg A, Nyström FH. A cross-over study of postprandial effects from moist snuff and red wine on metabolic rate, appetite-related hormones and glucose. Drug Alcohol Depend. 2022;236: 109479. 35580478 10.1016/j.drugalcdep.2022.109479

[5] Piano MR, Benowitz NL, Fitzgerald GA, Corbridge S, Heath J, Hahn E, Pechacek TF, Howard G, American Heart Association Council on Cardiovascular N. Impact of smokeless tobacco products on cardiovascular disease: implications for policy, prevention, and treatment: a policy statement from the American Heart Association. Circulation. 2010;122:1520–44. 20837898 10.1161/CIR.0b013e3181f432c3

[6] Omvik P. How smoking affects blood pressure. Blood Press. 1996;5:71–7. 9162447 10.3109/08037059609062111

[7] af Geijerstam P, Janryd F, Nyström FH. Smoking and cardiovascular disease in patients with type 2 diabetes: a prospective observational study. J Cardiovasc Med. 2023;24:802–7. 10.2459/JCM.0000000000001540 PMC10552835 37768866

[8] Kahn SE, Hull RL, Utzschneider KM. Mechanisms linking obesity to insulin resistance and type 2 diabetes. Nature. 2006;444:840–6. 17167471 10.1038/nature05482

[9] Chiolero A, Faeh D, Paccaud F, Cornuz J. Consequences of smoking for body weight, body fat distribution, and insulin resistance. Am J Clin Nutr. 2008;87:801–9. 18400700 10.1093/ajcn/87.4.801

[10] Audrain-McGovern J, Benowitz NL. Cigarette smoking, nicotine, and body weight. Clin Pharmacol Ther. 2011;90:164–8. 21633341 10.1038/clpt.2011.105 PMC3195407

[11] Hofstetter A, Schutz Y, Jéquier E, Wahren J. Increased 24-hour energy expenditure in cigarette smokers. N Engl J Med. 1986;314:79–82. 3941694 10.1056/NEJM198601093140204

[12] Herman RJ, Schmidt HD. Targeting GLP-1 receptors to reduce nicotine use disorder: preclinical and clinical evidence. Physiol Behav. 2024;281: 114565. 38663460 10.1016/j.physbeh.2024.114565 PMC11128349

[13] Titova OE, Baron JA, Michaelsson K, Larsson SC. Swedish snuff (snus) and risk of cardiovascular disease and mortality: prospective cohort study of middle-aged and older individuals. BMC Med. 2021;19:111. 33957912 10.1186/s12916-021-01979-6 PMC8103653

[14] Ling PM, Hrywna M, Talbot EM, Lewis MJ. Tobacco-derived nicotine pouch brands and marketing messages on internet and traditional media: content analysis. JMIR Form Res. 2023;7: e39146. 36790840 10.2196/39146 PMC9978966

[15] Tattan-Birch H, Jackson SE, Dockrell M, Brown J. Tobacco-free nicotine pouch use in Great Britain: a representative population survey 2020–2021. Nicotine Tob Res. 2022;24:1509–12. 35417551 10.1093/ntr/ntac099 PMC9356773

[16] Majmundar A, Okitondo C, Xue A, Asare S, Bandi P, Nargis N. Nicotine pouch sales trends in the US by volume and nicotine concentration levels from 2019 to 2022. JAMA Netw Open. 2022;5: e2242235. 36378312 10.1001/jamanetworkopen.2022.42235 PMC9667333

[17] Nahhas GJ, Cummings KM, Halenar MJ, Sharma E, Alberg AJ, Hatuskami D, Bansal-Travers M, Hyland A, Gaalema DE, Morris PB, et al. Smokeless tobacco use and prevalence of cardiovascular disease among males in the Population Assessment of Tobacco and Health (PATH) Study, waves 1–4. Prev Med Rep. 2022;25: 101650. 35127346 10.1016/j.pmedr.2021.101650 PMC8800067

[18] Byhamre ML, Araghi M, Alfredsson L, Bellocco R, Engström G, Eriksson M, Galanti MR, Jansson JH, Lager A, Lundberg M, et al. Swedish snus use is associated with mortality: a pooled analysis of eight prospective studies. Int J Epidemiol. 2021;49:2041–50. 33347584 10.1093/ije/dyaa197 PMC7825961

[19] Committee on Toxicity of Chemicals in Food CpatE: Statement on the bioavailability of nicotine from the use of oral nicotine pouches and assessment of the potential toxicological risk to users. The Food Standards Agency; 2023.

[20] Health risk assessment of nicotine pouches, Updated BfR Opinion no. 023/2022. Berlin, Germany: The German Federal Institute for Risk Assessment (BfR); 2022.

[21] Titova OE, Baron JA, Fall T, Michaelsson K, Larsson SC. Swedish snuff (Snus), cigarette smoking, and risk of type 2 diabetes. Am J Prev Med. 2023;65:60–6. 36754744 10.1016/j.amepre.2023.01.016

[22] Cleghorn CL, Harrison RA, Ransley JK, Wilkinson S, Thomas J, Cade JE. Can a dietary quality score derived from a short-form FFQ assess dietary quality in UK adult population surveys? Public Health Nutr. 2016;19:2915–23. 27181696 10.1017/S1368980016001099 PMC10271203

[23] Olsson SJ, Ekblom Ö, Andersson E, Börjesson M, Kallings LV. Categorical answer modes provide superior validity to open answers when asking for level of physical activity: a cross-sectional study. Scand J Public Health. 2016;44:70–6. 26392418 10.1177/1403494815602830

[24] Kallings LV, Olsson SJG, Ekblom Ö, Ekblom-Bak E, Börjesson M. The SED-GIH: a single-item question for assessment of stationary behaviour—a study of concurrent and convergent validity. Int J Environ Res Public Health. 2019;16:4766. 31795109 10.3390/ijerph16234766 PMC6926785

[25] Lancaster T, Stead LF. Individual behavioural counselling for smoking cessation. Cochrane Database Syst Rev. 2017;3:CD001292. 12137623 10.1002/14651858.CD001292

[26] Virtanen SE, Zeebari Z, Rohyo I, Galanti MR. Evaluation of a brief counseling for tobacco cessation in dental clinics among Swedish smokers and snus users. A cluster randomized controlled trial (the FRITT study). Prev Med. 2015;70:26–32. 25445335 10.1016/j.ypmed.2014.11.005

[27] Keller-Hamilton B, Alalwan MA, Curran H, Hinton A, Long L, Chrzan K, Wagener TL, Atkinson L, Suraapaneni S, Mays D. Evaluating the effects of nicotine concentration on the appeal and nicotine delivery of oral nicotine pouches among rural and Appalachian adults who smoke cigarettes: a randomized cross-over study. Addiction. 2024;119:464–75. 37964431 10.1111/add.16355 PMC10872395

[28] Mallock-Ohnesorg N, Rabenstein A, Stoll Y, Gertzen M, Rieder B, Malke S, Burgmann N, Laux P, Pieper E, Schulz T, et al. Small pouches, but high nicotine doses-nicotine delivery and acute effects after use of tobacco-free nicotine pouches. Front Pharmacol. 2024;15:1392027. 38841367 10.3389/fphar.2024.1392027 PMC11150668

[29] Martinez-Morata I, Sanchez TR, Shimbo D, Navas-Acien A. Electronic cigarette use and blood pressure endpoints: a systematic review. Curr Hypertens Rep. 2020;23:2. 33230755 10.1007/s11906-020-01119-0 PMC10867863

[30] Lee DH, Ha MH, Kim JR, Jacobs DR Jr. Effects of smoking cessation on changes in blood pressure and incidence of hypertension: a 4-year follow-up study. Hypertension. 2001;37:194–8. 11230270 10.1161/01.hyp.37.2.194

[31] Imai Y, Ohkubo T, Hozawa A, Tsuji I, Matsubara M, Araki T, Chonan K, Kikuya M, Satoh H, Hisamichi S, Nagai K. Usefulness of home blood pressure measurements in assessing the effect of treatment in a single-blind placebo-controlled open trial. J Hypertens. 2001;19:179–85. 11212959 10.1097/00004872-200102000-00003

[32] Moffatt RJ, Biggerstaff KD, Stamford BA. Effects of the transdermal nicotine patch on normalization of HDL-C and its subfractions. Prev Med. 2000;31:148–52. 10938215 10.1006/pmed.2000.0694

[33] Rao ChS, Subash YE. The effect of chronic tobacco smoking and chewing on the lipid profile. J Clin Diagn Res. 2013;7:31–4. 23449989 10.7860/JCDR/2012/5086.2663 PMC3576744

[34] Chelland Campbell S, Moffatt RJ, Stamford BA. Smoking and smoking cessation—the relationship between cardiovascular disease and lipoprotein metabolism: a review. Atherosclerosis. 2008;201:225–35. 18565528 10.1016/j.atherosclerosis.2008.04.046

[35] Asthana A, Johnson HM, Piper ME, Fiore MC, Baker TB, Stein JH. Effects of smoking intensity and cessation on inflammatory markers in a large cohort of active smokers. Am Heart J. 2010;160:458–63. 20826253 10.1016/j.ahj.2010.06.006 PMC2937015

[36] Hastie CE, Haw S, Pell JP. Impact of smoking cessation and lifetime exposure on C-reactive protein. Nicotine Tob Res. 2008;10:637–42. 18418786 10.1080/14622200801978722

[37] Mainous AG 3rd, Yadav S, Hong YR, Huo J. e-Cigarette and conventional tobacco cigarette use, dual use, and C-reactive protein. J Am Coll Cardiol. 2020;75:2271–3. 32354388 10.1016/j.jacc.2020.02.061

[38] Peres FS, Barreto SM, Camelo LV, Ribeiro ALP, Vidigal PG, Duncan BB, Giatti L. Time from smoking cessation and inflammatory markers: new evidence from a cross-sectional analysis of ELSA-Brasil. Nicotine Tob Res. 2017;19:852–8. 28164227 10.1093/ntr/ntx032

[39] Gallus S, Lugo A, Suatoni P, Taverna F, Bertocchi E, Boffi R, Marchiano A, Morelli D, Pastorino U. Effect of tobacco smoking cessation on C-reactive protein levels in a cohort of low-dose computed tomography screening participants. Sci Rep. 2018;8:12908. 30150729 10.1038/s41598-018-29867-9 PMC6110802

[40] Rohleder N, Kirschbaum C. The hypothalamic-pituitary-adrenal (HPA) axis in habitual smokers. Int J Psychophysiol. 2006;59:236–43. 16325948 10.1016/j.ijpsycho.2005.10.012

[41] Rothman KJ. No adjustments are needed for multiple comparisons. Epidemiology. 1990;1:43–6. 2081237

