# Supplementary material for: Cardiovascular and metabolic changes following 12 weeks of tobacco and nicotine pouch cessation: a Swedish cohort study
Source: Harm Reduct J. 2025 Apr 16;22:54. doi: 10.1186/s12954-025-01195-y (PMC12001473; doi:10.1186/s12954-025-01195-y)
Supplement: Supplementary file 1 — Additional file 1. [file 12954_2025_1195_MOESM1_ESM.docx]

Title: Cardiovascular and Metabolic Changes Following 12 Weeks of Tobacco and Nicotine Pouch Cessation: A Swedish Cohort Study

Authors (surnames underlined):

Peder af Geijerstam, ORCID 0000-0001-6038-5131 (a), Annelie Joelsson (b), Karin Rådholm, ORCID 0000-0003-3120-0913 (a, c), Fredrik H. Nyström, ORCID 0000-0002-1680-1000 (a)

Supplementary material

**Results**

Of 37 participants, the missing values until loss to follow up or completion of the study was 4414 (24%) for all home BP measurements and 541 (17%) for entire days of home BP measurements, mostly because 8 of the participants stopped reporting home BP values from week 5 (n = 2), 6 (n = 2), and 7-10 (n = 1 each). Missing values were 11 (10%) for body weight measurements, and 99 (9%) for laboratory values. Laboratory measurements were performed after a median of 28 (Q1-Q3 27-29) and 84 (Q1-Q3 83-87) days. One participant forgot to register home BP values week 12 (the last week), and instead registered them during week 13, and these BP values were used in place of week 12. Laboratory results for week 4 were censored for 2 participants because of delayed sampling. Finally, the Pearson correlation coefficient between the baseline systolic office and home BP was 0.87 (95% CI 0.77-0.93), *P* <.001.

In individuals using nicotine pouches during the run-in, but not in those using tobacco pouches, plasma cholesterol and LDL levels increased until week 12, mean (95% CI) 0.62 (0.28-0.96) and 0.64 (0.32-0.95) mmol/L difference from the run-in period, respectively, Supplementary table 2. This difference remained after adjustment for age, sex, and pouch years.

Finally, 6 (16%) participants were referred to their primary care center after study completion because they had previously unknown hypertension (n = 1) or pre-diabetes (n = 1) from run-in, and/or because they developed systolic hypertension (n = 2), pre-diabetes (n = 2), or elevated LDL levels (n = 1) during follow-up, Supplementary table 3.

**Supplementary figure 1.** Boxes of nicotine (left), dry tobacco (middle), and moist tobacco pouches (right), and their respective content. Photo: Peder af Geijerstam.


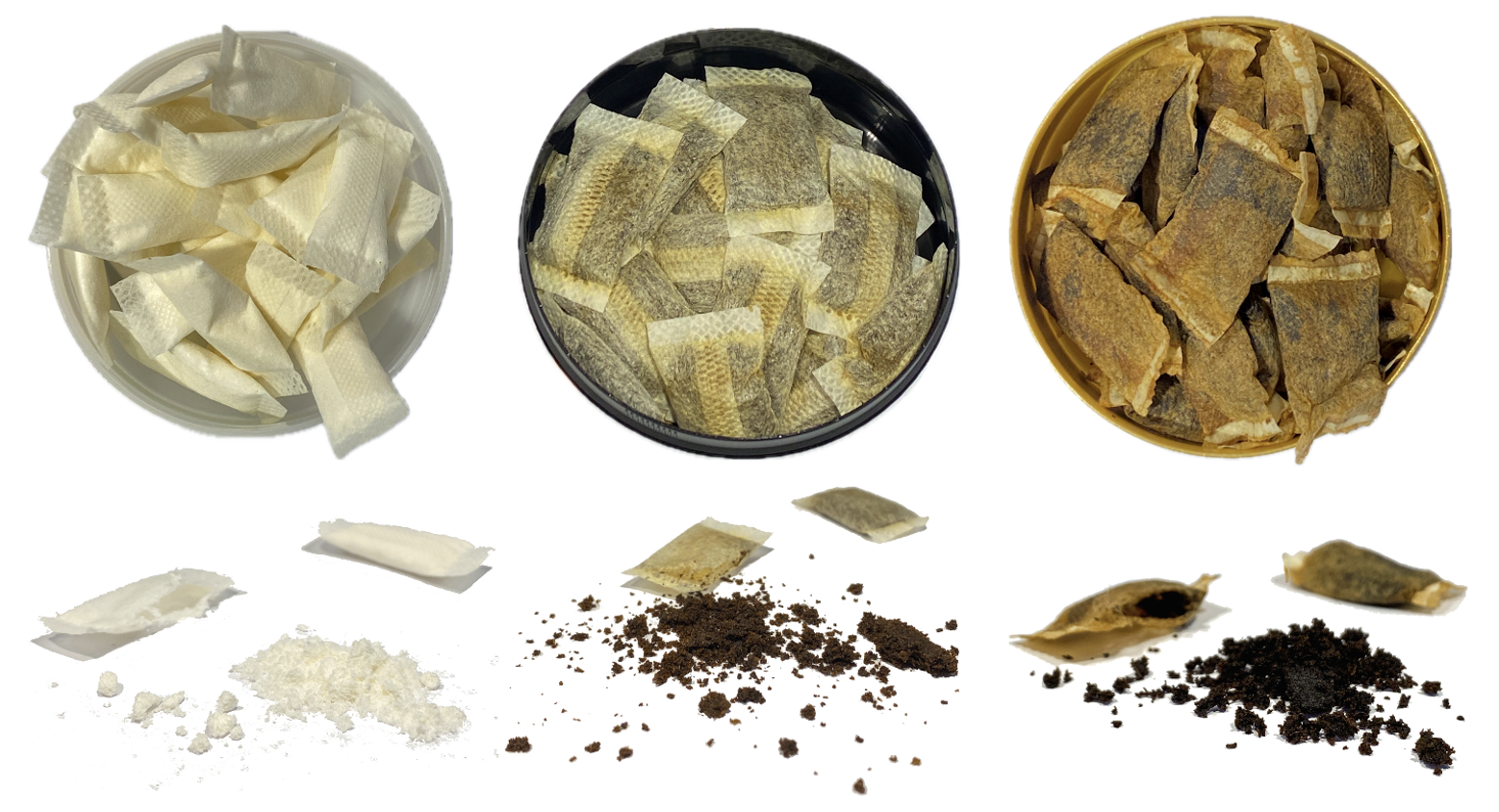


**Supplementary figure 2.** Study flow chart. Percentages are of the initial 50 individuals.


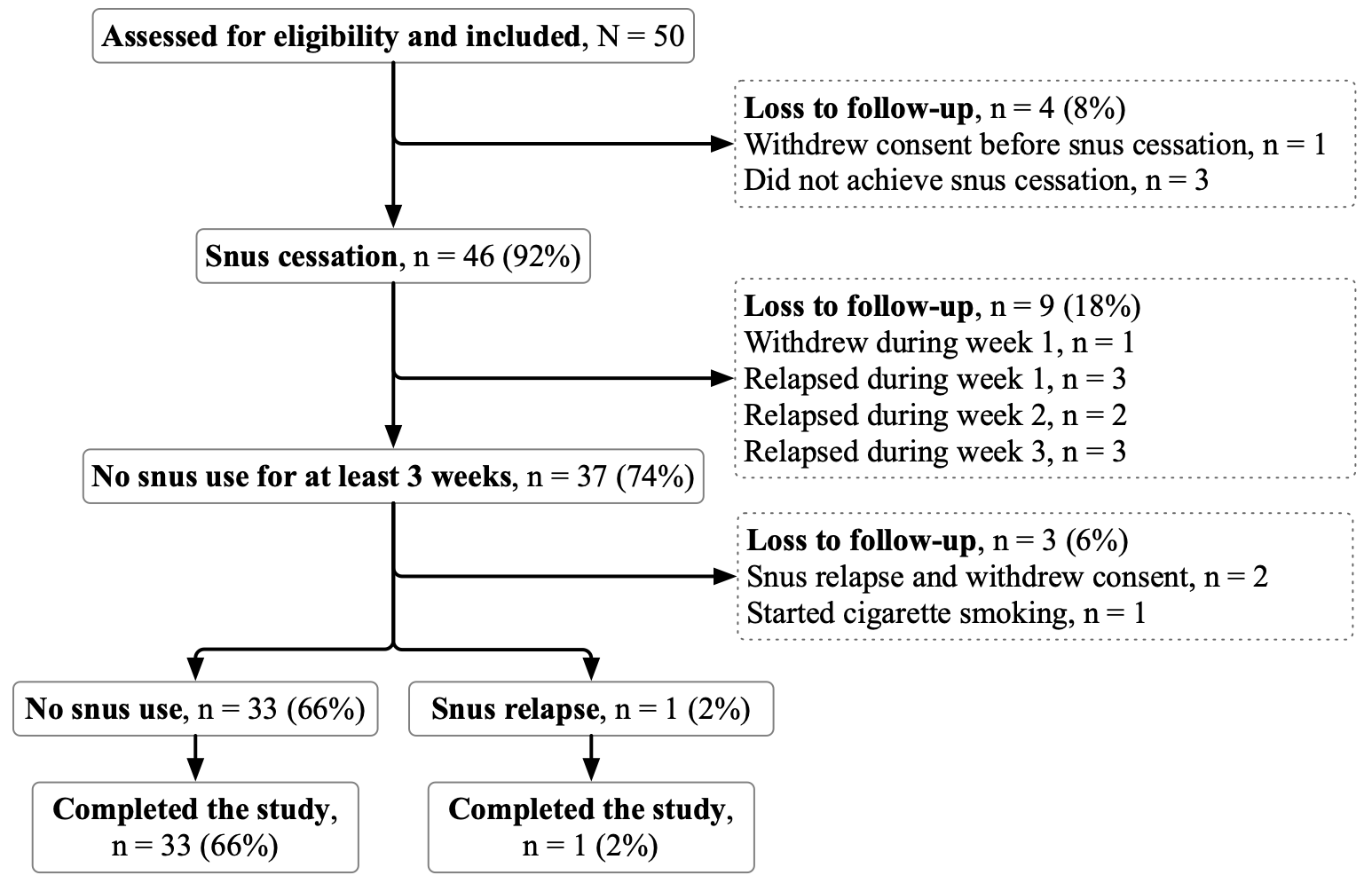


**Supplementary figure 3.** Daily nicotine intake during run-in in mg (A) and life-time snus use in pouch years (B).


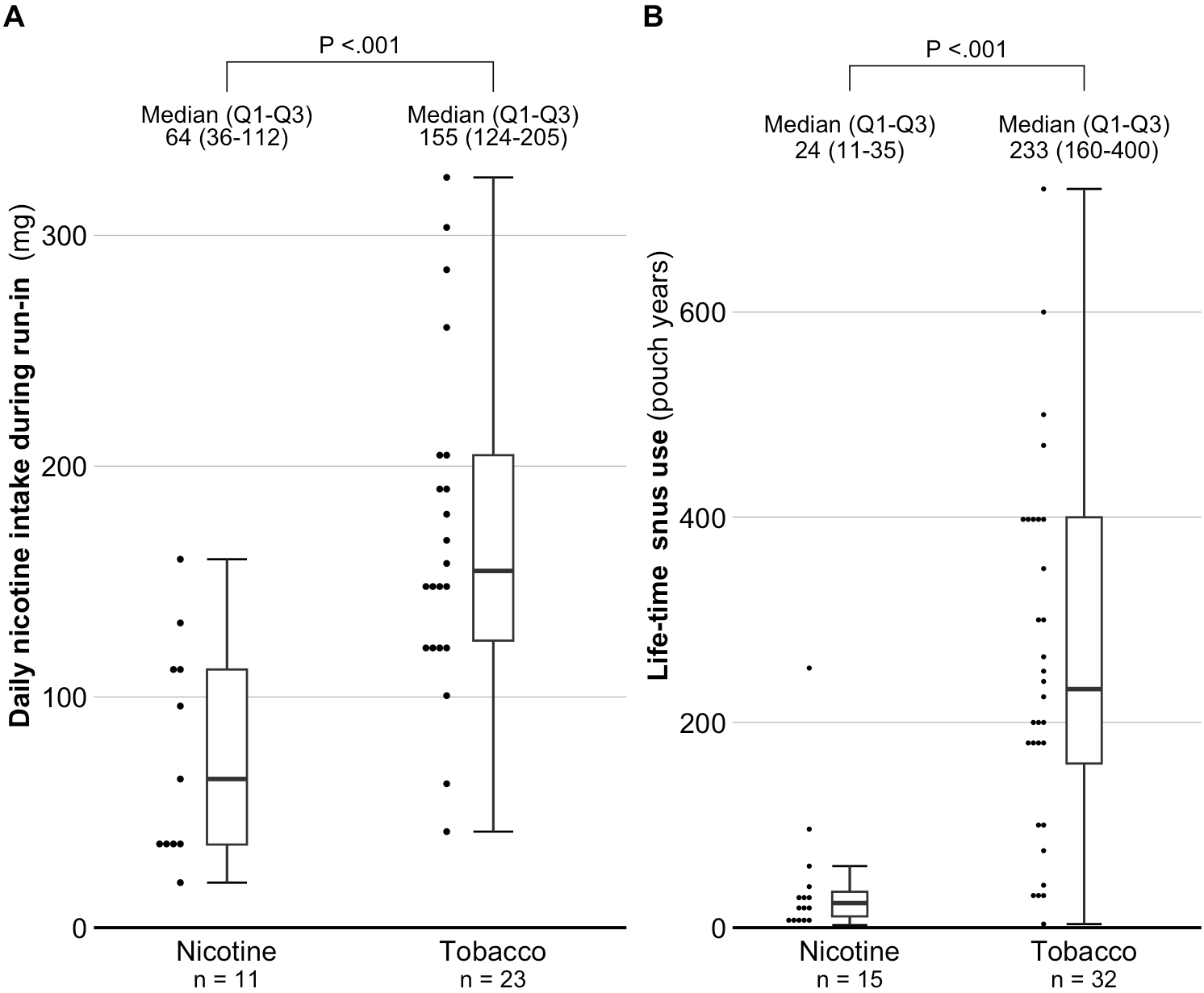


Snus pouch years were calculated as the years of consumption multiplied by the average number of pouches per day. For (A), one outlier with a nicotine intake of 637 mg per day during run-in is not shown, 1 participant is not included because they used both snus types, and 2 participants are not included because they did not define the number of pouches used during run-in. For (B), one outlier with 1200 pouch years is not shown. Comparisons were made using Wilcoxon rank-sum test.

**Supplementary table 1.** Results from the food frequency questionnaire and AUDIT during snus cessation.

|  |  | Run-in | Week 4 | Week 12 | Change |
| --- | --- | --- | --- | --- | --- |
|  | **N** | **Mean (95% CI)** | **Mean (95% CI)** | **Mean (95% CI)** | **Mean (95% CI)** |
| FFQ, total intake (grams) |  |  |  |  |  |
| Run-in to Week 4 | 35 | 514.3 (444.4-584.1) | 470.6 (381.4-559.7) |  | -43.7 (-131.4 to 44.1) |
| Week 4 to Week 12 | 32 |  | 492.7 (401.0-584.4) | 470.2 (378.8-561.6) | -22.5 (-138.4 to 93.4) |
| Run-in to Week 12 | 34 | 516.1 (445.9-586.4) |  | 459.5 (369.0-550.0) | -56.6 (-155.6 to 42.4) |
| FFQ, fat intake (grams) |  |  |  |  |  |
| Run-in to Week 4 | 35 | 67.8 (54.7-80.9) | 61.5 (48.8-74.2) |  | -6.3 (-22.8 to 10.2) |
| Week 4 to Week 12 | 32 |  | 65.2 (52.2-78.3) | 56.5 (46.4-66.5) | -8.8 (-21.9 to 4.4) |
| Run-in to Week 12 | 34 | 69.1 (55.9-82.4) |  | 54.7 (44.7-64.7) | -14.5 (-29.4 to 0.5) |
| FFQ, sugars intake (grams) |  |  |  |  |  |
| Run-in to Week 4 | 35 | 36.4 (24.9-47.9) | 31.2 (24.2-38.3) |  | -5.1 (-16.9 to 6.6) |
| Week 4 to Week 12 | 32 |  | 32.1 (24.7-39.6) | 26.6 (19.8-33.4) | -5.6 (-12.8 to 1.7) |
| Run-in to Week 12 | 34 | 36.3 (24.6-47.9) |  | 26.5 (19.8-33.2) | -9.8 (-21.6 to 2.0) |
| AUDIT, total score (points) |  |  |  |  |  |
| Run-in to Week 4 | 37 | 2.3 (1.5-3.2) | 2.2 (1.4-3.0) |  | -0.1 (-0.6 to 0.3) |
| Week 4 to Week 12 | 37 |  | 2.2 (1.4-3.0) | 1.9 (1.0-2.8) | -0.3 (-0.8 to 0.3) |
| Run-in to Week 12 | 37 | 2.3 (1.5-3.2) |  | 2.0 (1.1-2.8) | -0.4 (-0.9 to 0.2) |

Differences between each week of snus cessation and the run-in were made using a 2-sided paired Wilcoxon signed-rank test and presented as the mean (95% CI) difference and the *P* value. Abbreviations: AUDIT, Alcohol use disorders identification test; FFQ, food frequency questionnaire.

**Supplementary table 2.** Subgroup analysis of changes in body weight and blood sample values for participants using nicotine and tobacco pouches during run-in. One participant used both types of pouches and is not shown. Adjustments were for age, sex, and pouch years.

|  | Nicotine pouches, crude | | Tobacco pouches, crude | | Difference | Nicotine pouches, adjusted | Tobacco pouches, adjusted | Difference |
| --- | --- | --- | --- | --- | --- | --- | --- | --- |
|  | **n** | **Mean (95% CI)** | **n** | **Mean (95% CI)** | **Mean (95% CI)** | **Mean (95% CI)** | **Mean (95% CI)** | **Mean (95% CI)** |
| Body weight (kg) |  |  |  |  |  |  |  |  |
| Run-in to Week 4 | 11 | 1.5 (0.8 to 2.3) | 21 | 2.0 (1.4 to 2.5) | -0.5 (-1.4 to 0.5) | 1.7 (0.7 to 2.6) | 1.7 (1.0 to 2.4) | -0.0 (-1.3 to 1.3) |
| Week 4 to Week 12 | 11 | 0.1 (-0.9 to 1.2) | 18 | 0.2 (-0.6 to 1.0) | -0.1 (-1.4 to 1.2) | -0.3 (-1.5 to 0.9) | 0.7 (-0.3 to 1.7) | -1.0 (-2.7 to 0.8) |
| Run-in to Week 12 | 11 | 1.6 (0.5 to 2.8) | 20 | 1.9 (1.0 to 2.7) | -0.2 (-1.6 to 1.2) | 1.1 (-0.2 to 2.4) | 2.3 (1.3 to 3.3) | -1.2 (-3.1 to 0.6) |
| Plasma total cholesterol (mmol/L) |  |  |  |  |  |  |  |  |
| Run-in to Week 4 | 11 | 0.39 (0.12 to 0.66) | 22 | 0.13 (-0.06 to 0.32) | 0.26 (-0.07 to 0.60) | 0.43 (0.10 to 0.76) | 0.12 (-0.13 to 0.37) | 0.31 (-0.15 to 0.77) |
| Week 4 to Week 12 | 11 | 0.18 (-0.11 to 0.47) | 19 | -0.16 (-0.39 to 0.06) | 0.34 (-0.02 to 0.71) | 0.27 (-0.08 to 0.62) | -0.23 (-0.51 to 0.04) | 0.50 (0.00 to 1.01) |
| Run-in to Week 12 | 11 | 0.57 (0.30 to 0.85) | 20 | -0.05 (-0.26 to 0.16) | 0.62 (0.28 to 0.97) | 0.62 (0.28 to 0.96) | -0.08 (-0.34 to 0.19) | 0.70 (0.21 to 1.18) |
| Plasma triglycerides (mmol/L) |  |  |  |  |  |  |  |  |
| Run-in to Week 4 | 11 | 0.01 (-0.21 to 0.22) | 22 | 0.09 (-0.06 to 0.24) | -0.09 (-0.35 to 0.17) | 0.02 (-0.23 to 0.27) | 0.03 (-0.16 to 0.22) | -0.01 (-0.36 to 0.34) |
| Week 4 to Week 12 | 11 | -0.10 (-0.32 to 0.13) | 19 | -0.00 (-0.17 to 0.17) | -0.10 (-0.38 to 0.19) | -0.10 (-0.38 to 0.18) | 0.00 (-0.22 to 0.23) | -0.10 (-0.51 to 0.30) |
| Run-in to Week 12 | 11 | -0.09 (-0.35 to 0.17) | 20 | 0.01 (-0.18 to 0.20) | -0.10 (-0.43 to 0.22) | -0.18 (-0.49 to 0.13) | 0.03 (-0.21 to 0.27) | -0.21 (-0.65 to 0.22) |
| Plasma LDL (mmol/L) |  |  |  |  |  |  |  |  |
| Run-in to Week 4 | 11 | 0.31 (0.07 to 0.55) | 22 | 0.05 (-0.12 to 0.21) | 0.26 (-0.03 to 0.55) | 0.30 (0.02 to 0.59) | 0.09 (-0.12 to 0.31) | 0.21 (-0.19 to 0.61) |
| Week 4 to Week 12 | 11 | 0.23 (-0.05 to 0.50) | 19 | -0.16 (-0.37 to 0.04) | 0.39 (0.05 to 0.73) | 0.34 (0.01 to 0.66) | -0.26 (-0.52 to 0.00) | 0.60 (0.13 to 1.06) |
| Run-in to Week 12 | 11 | 0.54 (0.28 to 0.80) | 20 | -0.07 (-0.26 to 0.12) | 0.61 (0.28 to 0.93) | 0.64 (0.32 to 0.95) | -0.13 (-0.37 to 0.12) | 0.76 (0.32 to 1.21) |
| Plasma HDL (mmol/L) |  |  |  |  |  |  |  |  |
| Run-in to Week 4 | 11 | 0.08 (0.02 to 0.14) | 22 | 0.04 (0.00 to 0.09) | 0.04 (-0.04 to 0.11) | 0.09 (0.01 to 0.16) | 0.05 (-0.00 to 0.11) | 0.03 (-0.07 to 0.13) |
| Week 4 to Week 12 | 11 | -0.02 (-0.10 to 0.07) | 19 | -0.02 (-0.08 to 0.04) | 0.01 (-0.10 to 0.11) | -0.03 (-0.13 to 0.08) | -0.01 (-0.09 to 0.08) | -0.02 (-0.17 to 0.13) |
| Run-in to Week 12 | 11 | 0.07 (-0.02 to 0.16) | 20 | 0.01 (-0.05 to 0.08) | 0.05 (-0.06 to 0.16) | 0.05 (-0.06 to 0.15) | 0.04 (-0.04 to 0.13) | 0.00 (-0.15 to 0.15) |
| Plasma non-HDL (mmol/L) |  |  |  |  |  |  |  |  |
| Run-in to Week 4 | 11 | 0.34 (0.09 to 0.59) | 22 | 0.09 (-0.09 to 0.26) | 0.25 (-0.05 to 0.55) | 0.34 (0.03 to 0.64) | 0.09 (-0.14 to 0.32) | 0.25 (-0.18 to 0.67) |
| Week 4 to Week 12 | 11 | 0.16 (-0.12 to 0.45) | 19 | -0.15 (-0.36 to 0.07) | 0.31 (-0.04 to 0.66) | 0.28 (-0.06 to 0.61) | -0.24 (-0.51 to 0.03) | 0.51 (0.03 to 1.00) |
| Run-in to Week 12 | 11 | 0.50 (0.25 to 0.75) | 20 | -0.06 (-0.25 to 0.13) | 0.56 (0.24 to 0.88) | 0.55 (0.24 to 0.87) | -0.10 (-0.34 to 0.14) | 0.66 (0.22 to 1.10) |
| Plasma glucose (mmol/L) |  |  |  |  |  |  |  |  |
| Run-in to Week 4 | 11 | 0.02 (-0.20 to 0.23) | 22 | 0.01 (-0.14 to 0.16) | 0.01 (-0.25 to 0.27) | 0.03 (-0.23 to 0.30) | -0.00 (-0.20 to 0.20) | 0.04 (-0.34 to 0.41) |
| Week 4 to Week 12 | 11 | -0.01 (-0.17 to 0.16) | 19 | 0.14 (0.01 to 0.26) | -0.15 (-0.35 to 0.06) | -0.04 (-0.24 to 0.16) | 0.15 (-0.01 to 0.32) | -0.19 (-0.49 to 0.10) |
| Run-in to Week 12 | 11 | 0.01 (-0.22 to 0.24) | 20 | 0.12 (-0.06 to 0.29) | -0.11 (-0.39 to 0.18) | -0.04 (-0.32 to 0.25) | 0.15 (-0.07 to 0.37) | -0.19 (-0.59 to 0.21) |
| Blood HbA1c (mmol/mol) |  |  |  |  |  |  |  |  |
| Run-in to Week 4 | 11 | -0.3 (-1.3 to 0.8) | 22 | 0.2 (-0.6 to 0.9) | -0.5 (-1.8 to 0.8) | -0.72 (-1.99 to 0.54) | 0.60 (-0.36 to 1.55) | -1.32 (-3.09 to 0.45) |
| Week 4 to Week 12 | 11 | 0.9 (-0.4 to 2.2) | 19 | 0.5 (-0.5 to 1.4) | 0.4 (-1.2 to 2.0) | 1.36 (-0.17 to 2.88) | 0.29 (-0.92 to 1.51) | 1.06 (-1.12 to 3.25) |
| Run-in to Week 12 | 11 | 0.6 (-0.6 to 1.9) | 20 | 0.8 (-0.1 to 1.7) | -0.2 (-1.7 to 1.4) | 0.80 (-0.70 to 2.30) | 0.92 (-0.23 to 2.08) | -0.12 (-2.22 to 1.99) |
| Plasma insulin (mIE/L) |  |  |  |  |  |  |  |  |
| Run-in to Week 4 | 11 | 1.48 (-0.43 to 3.39) | 22 | 0.05 (-1.30 to 1.39) | 1.44 (-0.90 to 3.77) | 0.20 (-1.88 to 2.29) | 0.73 (-0.84 to 2.30) | -0.53 (-3.44 to 2.39) |
| Week 4 to Week 12 | 11 | 0.17 (-2.12 to 2.47) | 19 | -0.47 (-2.22 to 1.28) | 0.64 (-2.25 to 3.53) | 0.71 (-2.12 to 3.54) | -0.93 (-3.18 to 1.32) | 1.64 (-2.41 to 5.70) |
| Run-in to Week 12 | 11 | 1.65 (-0.62 to 3.93) | 20 | -0.73 (-2.41 to 0.95) | 2.38 (-0.44 to 5.21) | 0.82 (-1.70 to 3.33) | -0.34 (-2.28 to 1.59) | 1.16 (-2.37 to 4.69) |
| Plasma hsCRP (mg/L) |  |  |  |  |  |  |  |  |
| Run-in to Week 4 | 10 | 0.13 (-0.63 to 0.89) | 22 | 0.61 (0.09 to 1.12) | -0.47 (-1.39 to 0.44) | 0.20 (-0.72 to 1.13) | 0.64 (-0.03 to 1.31) | -0.44 (-1.69 to 0.81) |
| Week 4 to Week 12 | 10 | -0.15 (-0.57 to 0.27) | 19 | -0.21 (-0.52 to 0.09) | 0.06 (-0.46 to 0.58) | 0.04 (-0.46 to 0.54) | -0.33 (-0.71 to 0.05) | 0.37 (-0.32 to 1.07) |
| Run-in to Week 12 | 11 | -0.02 (-0.74 to 0.70) | 20 | 0.40 (-0.14 to 0.93) | -0.42 (-1.31 to 0.48) | 0.20 (-0.65 to 1.06) | 0.31 (-0.35 to 0.97) | -0.11 (-1.31 to 1.09) |

**Supplementary table 3.** Participants referred to their primary care center for follow-up.

|  | Baseline | Week 4 | Week 12 |
| --- | --- | --- | --- |
| Hypertension, systolic home blood pressure (mmHg) |  |  |  |
| 30 to 40-year old man | 131 | 131 | 138 |
| 50 to 60-year old man | 148 | 162 | N/A^a^ |
| 60 to 70-year old man | 135 | 135 | 136 |
| Pre-diabetes, fP-glucose (mmol/L) |  |  |  |
| 30 to 40-year old man | 6.4 | 6.3 | 6.2 |
| 30 to 40-year old man | 5.6 | 6.1 | 6.3 |
| Elevated low-density lipoprotein (mmol/L) |  |  |  |
| 30 to 40-year old man | 4.6 | 4.1 | 5.1 |

a. Already referred and on treatment, and thus not measured.
